# Genome Sequence and Characteristics of Cluster AS3 *Arthrobacter globiformis* Phages Atlantica, Babushka, DanHam62, and Glotell

**DOI:** 10.17912/micropub.biology.002013

**Published:** 2026-07-21

**Authors:** Sarah Bakayoko, Justin Chen, Yu-Chuan Chen, Valerie N. Jackson, Jacob Hillman, Daniela Jara, Leah Joby, Ramanpreet Kaur, Nikhita Lalwani, Kirsty Larsen, Loren Lewis, Martin Lin, Hameeda Rasheed, Faizan Tariq, Chennai Wallis, Bryan Gibb, James T. Melton III, Nic M. Vega

**Affiliations:** 1 Department of Biological and Chemical Sciences, New York Institute of Technology, Old Westbury, NY, United States; 2 Department of Biology, Emory University, Atlanta, GA, United States; 3 Department of Biology, Spelman College, Atlanta, GA, United States

## Abstract

We report the discovery and characterization of four phages infecting *Arthrobacter globiformis* B-2979 that are all assigned to actinobacteriophage subcluster AS3 based on gene content similarity. These phages have genomes of approximately 38 kbp, with 66-71 predicted protein-coding genes and no tRNA genes. Genome architecture suggests that these phage are temperate. An *attP* core sequence with homology to host tRNA-fMet has been identified.

**Figure 1. Traits of four cluster AS3 phage:** Plaque morphology (A-D) and negative-staining TEM (E-H) with 1% uranyl acetate of phage Atlantica (A, E), Babushka (B, F), DanHam62 (C, G), and Glotell (D, H). Plaque formation on isolation host B-2979 with 0.2% (Atlantica) or 0.4% PYCa top agar after 24 hours (Atlantica) or 48 hours (Babushka, DanHam62, Glotell) at 30°C. Imaging was carried out at each originating institution (Atlantica, Emory University; Babushka and DanHam62, NYIT; Glotell, Spelman University). (I) Alignment of the putative *attP* site and upstream region on the phage Atlantica genome with the corresponding regions on the genomes of Babushka, DanHam62, and Glotell. The homologous region on the genome of phage Melons (AS2) and the putative *attP* site of phage Galaxy (AS1; genome coordinates 20,716–20,755) are shown to illustrate conservation. Multi-sequence alignment was carried out using NCBI BLAST with default parameters for somewhat similar sequences ( *blastn* ), and the alignment was visualized in Jalview 2.11.5.1. Table: Isolation and characterization of AS3 phage Atlantica, Babushka, DanHam62, and Glotell. Protocols were carried out using the equipment and resources at each originating institution (Atlantica, Emory University; Babushka and DanHam62, NYIT; Glotell, Spelman University).

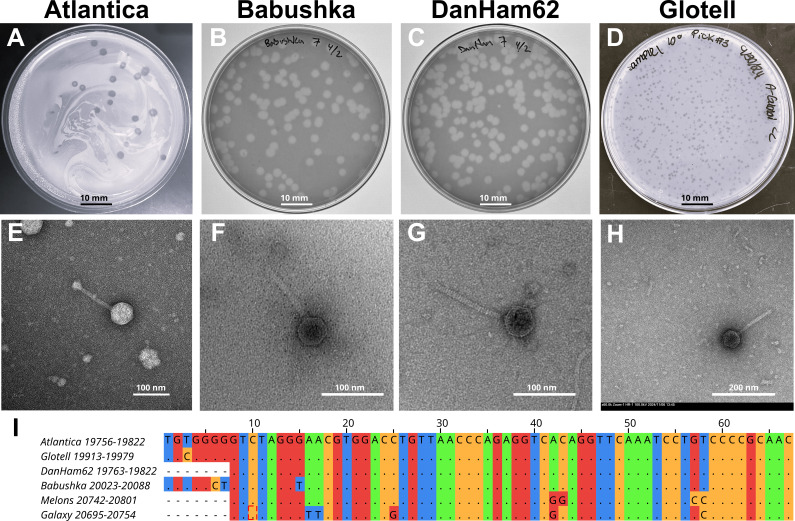



**Table:** 

A. globiformis Phage	Atlantica	Babushka	DanHam62	Glotell
Soil sample	Sandy, dry soil outside a garden	Moist soil from a backyard	Moist soil from a backyard	Dry soil/clay sample from a backyard
Location Found	Atlanta, GA	New York, NY	New York, NY	Atlanta, GA
Latitude	33.79672 N	40.73135 N	40.73652 N	33.29193 N
Longitude	84.32394 W	73.72391 W	73.70929 W	84.7119 W
Environment Temp (C)	23	25	25	N/A
Plaque Diameter (mm, range)	1-2 (n=8)	2-6 (n-10)	2-6 (n=10)	0.5-1.5 (n=30)
Tail length (nm, mean ± SD)	134 ± 3.5 (n=4)	126.4 ± 2.3 (n=3)	124.31 ± 3.9 (n=3)	131.76 ± 2.36 (n=3)
Capsid Diameter	62 ± 1.5 (n=5)	56.14 ± 1.8 (n=3)	50.1 ± 1.8 (n=3)	61.76 ± 0.70 (n=3)
DNA Isolation	Based on (Sobolewski et al., 2022)	Promega Wizard DNA Clean-Up kit	Promega Wizard DNA Clean-Up kit	Promega Wizard DNA Clean-Up kit
Number of Reads	3.7 million	1.5 million	1.25 million	0.98 million
Sequencing Coverage	9,427	3,677	3,130	2,501
Genome Size (bp)	38,468	38,467	38,380	38,515
Genome %GC	66.1	66	66	66.1
Number of genes	69	66	69	71
Number of genes with putative functions	43 (63%)	43 (65%)	45 (65%)	39 (54.9%)
Genome Accession	PX136956	PV915904	PV940979	PV876937
SRA	SRX28484005	SRX28484006	SRX28484042	SRX28484005

## Description

Temperate bacteriophage, which can propagate through vertical transmission with the host, are common, with prophages being detectable in a substantial fraction of bacterial genomes (Tuttle & Buchan, 2020). Temperate phage can substantially affect the ecology and evolution of bacterial populations (Howard-Varona et al., 2017). Here we present the isolation and genome characterization of four genetically similar bacteriophages infecting the common soil actinobacterium Arthrobacter globiformis (Conn, 1948). All four are predicted to be temperate, and one has been demonstrated to establish lysogeny. The data collectively contribute toward a better understanding of phage diversity in this clade of hosts (Hatfull, 2020) and of temperate phage diversity more broadly.

The phages Atlantica, Babushka, DanHam62, and Glotell were extracted from soil samples collected in New York and Georgia, USA (Table 1) as part of the Science Education Alliance-Phage Hunters Advancing Genomics and Evolutionary Science (SEA-PHAGES) program (Jordan et al., 2014) and using common procedures (Zorawik et al., 2024). Briefly, soil samples were washed with peptone-yeast extract-calcium (PYCa) liquid medium and sterile-filtered (0.22 µM pore size). Filtrate was inoculated with A. globiformis B-2979 and incubated with shaking at 200 RPM and 30°C for 48h before centrifugation to pellet bacteria and sterile filtration of supernatant. To create double-layer plates, soft (0.2-0.4%) top agar containing A. globiformis B-2979 and aliquots of filtrates was overlaid on PYCa base agar and incubated for 24-48h at 30°C before viewing plaques. The morphology and diameter of plaques are presented in Table 1 and Figure 1, respectively. Replication of phages produced small to medium-sized, clear to hazy plaques ( Fig. 1, Table 1). Phage were purified by 2-3 rounds of picking a single, well-isolated plaque, followed by serial dilution and plating to determine homogeneity. Negative-staining transmission electron microscopy of all phages indicated a short-tailed siphovirus morphology with a nearly isocahedral capsid, consistent with other Caudoviricetes bacteriophages such as phage λ (Fig. 1). Measurements for capsid diameter and tail length can be found in Table 1.

Genomic DNA was purified from high-titer lysates of the purified phages using methods shown in Table 1. Illumina libraries were prepared with the NEB Ultra II FS kit and sequenced on an Illumina NextSeq 1000 (XLEP-P1 kit, single-end 100-bp reads) (Russell, 2018). Glotell was sequenced as part of a DOGEMS (Deconvolution of Genomes after En Masse Sequencing) sample (Russell, 2024). Raw reads were trimmed with cutadapt 4.7 (using the option: –nextseq-trim 30) and filtered with skewer 0.2.2 (using the options: -q 20 -Q 30 -n -l 50) prior to assembly. Trimmed reads were assembled per Russell et al. (Russell, 2018), using Newbler v2.9 (Miller et al., 2010) with default parameters, generating single contigs that were verified for completeness and genomic termini using Consed v2.9 (Gordon et al., 1998). The resulting genomes (Table 1) were ~38 kbp. All phages had termini characterized by 12-bp 3' single-stranded overhangs, and GC content was ~66%, similar to that of the host. Phages were assigned to subcluster AS3 based on gene content similarity of at least 35% to phages in the Actinobacteriophage Database (PhagesDB.org) (Pope et al., 2017; Russell & Hatfull, 2017).

The genomes were automatically annotated using DNA Master v5.23.6, build 270 (http://cobamide2.bio.pitt.edu/computer.htm) (Pope & Jacobs-Sera, 2018) and PECAAN v20241104 (discover.kbrinsgd.org) (Rinehart et al., 2016). The initial auto-annotation was performed with Glimmer v3.02b (Delcher et al., 2007) and GeneMark v2 or v4.28 (Besemer et al., 2001; Besemer & Borodovsky, 2005), along with Starterator (http://phages.wustl.edu/starterator/) to help refine predicted gene coordinates. BLAST (Altschul et al., 1990) using the NCBI nonredundant and actinobacteriophage databases, HHPred v2.0.13 (Söding et al., 2005) using PDB_mmCIF70, SCOPe70, Pfam-A, NCBI_Conserved_Domains (CD_v3.19) (Zimmermann et al., 2018), and Phamerator [Actino_Draft v595 database] (Cresawn et al., 2011) were used as comparative tools to identify the putative gene functions. No tRNAs were identified using Aragorn v1.2.41 (Laslett & Canback, 2004) and tRNAscan-SE v2.0.12 (Lowe & Eddy, 1997). To assess the presence of transmembrane proteins, TMHMM v2.0 (Krogh et al., 2001) and TOPCONS 2.0 (Tsirigos et al., 2015) programs were used. Sequence alignments were created with BLASTN and visualized in Jalview 2.11.5.1 (Waterhouse et al., 2009). All software programs used default settings, unless otherwise specified.

All genomes were approximately 38.5 kb in length (Table 1) with a 3' sticky overhang at the genome ends (12 bp, GAGTTGCCGGCA). The resulting annotations indicated a λ-like genome architecture, with lysogeny-related genes and early genes transcribed on opposite sides of a putative bi-directional promoter (Echols & Murialdo, 1978). These genomes contained 66-71 predicted protein-coding genes, mostly transcribed in a single direction (~80% of genes) with the exception of a short block of genes near the middle of the genome (gp24 to gp36-39). Approximately 60% of genes could be assigned putative functions, including structural genes, lysis genes (endolysin and holin), and genes involved in DNA replication (RecE-like, RecT-like, RepA-like, helicase loader, SSB). Genes involved in lysogeny (Int-Y, immunity regulator) were among the alternate-strand genes, with exit-related genes (cro-like protein, excise) immediately downstream, reading in the primary direction. No tRNAs were present. One orpham was identified in the genome of Babushka (stop 37,513).

A region with 38 bp conserved homology to the 3' end of one of two tandem copies of *Arthrobacter* tRNA-fMet was identified between hypothetical proteins gp27 and gp28 on phage Atlantica (coordinates 19,783–19,821), several ORFs upstream of Int-Y (Atlantica gp36). BLASTN confirmed that this region is highly conserved within cluster AS ( Fig. 1I ) and is homologous to the putative *attP* site for the related *A. globiformis* phage Galaxy from subcluster AS1 (Klyczek et al., 2017). Integration of Atlantica at the proposed site was confirmed by PCR (Jackson & Vega, 2025).

**Nucleotide sequence accession numbers**

Complete genome sequences and sequence read archives (SRA) are available at GenBank. Accession numbers and SRA numbers are given in Table 1.

## Methods

N/A

## Reagents

N/A
